# Stabilization of the Surface of ZnO Films and Elimination of the Aging Effect

**DOI:** 10.3390/ma14216535

**Published:** 2021-10-30

**Authors:** Khabibulla A. Abdullin, Maratbek T. Gabdullin, Sultan K. Zhumagulov, Guzal A. Ismailova, Lesya V. Gritsenko, Yevgeniya Y. Kedruk, Mojtaba Mirzaeian

**Affiliations:** 1 Department of Physics and Technology, Al-Farabi Kazakh National University, Al-Farabi Ave. 71, Almaty 050040, Kazakhstan; zhumagulov.sultanbek@gmail.com (S.K.Z.); guzal_a81@mail.ru (G.A.I.); 2 Institute of Applied Science & Information Technology, Shashkin Str. 40–48, Almaty 050040, Kazakhstan; 3 Kazakh-British Technical University, Almaty 050000, Kazakhstan; gabdullin@physics.kz; 4 School of General Education, Satbayev University, Almaty 050013, Kazakhstan; gritsenko_lv@mail.ru (L.V.G.); y.kedruk@satbayev.university (Y.Y.K.); 5 School of Computing, Engineering and Physical Sciences, University of the West of Scotland, Paisley PA1 2BE, UK; mojtaba.mirzaeian@uws.ac.uk

**Keywords:** ZnO, chemical bath deposition, hydrogen plasma treatment, aging effect, surface stabilization

## Abstract

Zinc oxide is a promising multifunctional material. The practical use of nano- and polycrystalline ZnO devices faces a serious problem of instability of electrical and luminescent characteristics, due to the adsorption of oxygen by the surface during aging. In this paper, the aging effect in ZnO films and nanorod arrays was studied. It was found that ZnO samples demonstrate different behavior of the degradation process, which corresponds to at least two different types of adsorbing surface sites for O_2_, where O_2_ adsorption is of a different nature. The first type of surface sites is rapidly depassivated after hydrogen passivation and the aging effect takes place due to these centers. The second type of surface sites has a stable structure after hydrogen passivation and corresponds to HO–ZnO sites. The XPS components of these sites include the Zn2p_3/2_ peak at 1022.2 ± 0.2 eV and Zn2p_1/2_ peak at 1045.2 ± 0.2 eV, with a part of the XPS O1s peak at 531.5 ± 0.3 eV. The annealing transforms the first type of site into the second one, and the subsequent short-term plasma treatment in hydrogen results in steady passivation, where the degradation of characteristics is practically reduced to zero.

## 1. Introduction

Zinc oxide is an n-type conductivity semiconductor with a wide band gap of 3.37 eV, strong luminescence at room temperature, high conductivity and optical transparency [1,2]. Due to its unique electrical, optical, luminescent and catalytic properties, ZnO is a promising material for various applications [3], including chemical sensors [4,5], transparent field effect transistors [6], transparent conducting oxide [7], atomic force microscopy cantilever [8], nanopiezoelectric devices [9], UV detectors [10], etc. [11,12]. 

It is known that the luminescence, optical, and electrical properties of ZnO device structures are largely determined by the surface states and effects that take place on the surface of nano- and microcrystallites of ZnO films, nanorod arrays and nanoparticles [2,3,4,5,7]. For example, the photoluminescence (PL) intensity of the near band emission (NBE) is determined by the surface traps [13]; if the concentration of adsorbed species and surface states is controlled, the deep level emission (DLE) in the visible spectral range of ZnO nanoparticles can be completely inhibited [14]. 

The electrical properties of ZnO structures are also determined by surface effects [15,16]. Sheet resistance can be reduced by several orders of magnitude, due to the persistent photoconductivity effect, while resistivity is restored to high values after turning off UV radiation during the storage of samples under ambient conditions [17]. It has been shown that plasma treatment under a hydrogen atmosphere is an effective method for improving the electrical properties of ZnO films [18,19,20]. According to Dong et al., free carrier concentration and mobility in the ZnO can be varied from 1 × 10^17^ cm^−3^ and 7 cm^2^V^−1^s^−1^ in as-synthesized film up to 1 × 10^18^ cm^−3^ and 39 cm^2^V^−1^s^−1^ in the film treated in hydrogen plasma [19]. This effect is associated with the formation of hydrogen-related donor states, as well as with the hydrogen passivation of defects at grain boundaries [21]. However, the stability of this plasma treatment effect is quite high only in ZnO films synthesized at high temperatures (i.e., T > 400 °C) [18,21] and therefore, special treatment methods are required to ensure the high conductivity of ZnO films obtained by low temperature methods. 

Long-term performance stability is a critical general requirement for any device structure. Since the bulk properties of polycrystalline ZnO layers, nanorod arrays, and nanoparticles strongly depend on the surface states, it is very important to stabilize their surface properties. The practical application of nanostructured and polycrystalline ZnO is complicated by the fact that the performance of non-encapsulated ZnO layers does not have long-term stability since the surface is modified during storage under ambient conditions or under cyclic exposure to an oxidizing atmosphere and humidity as a result of which the bulk properties are subject to strong variations with shelf aging in atmospheric oxygen [22,23,24,25]. However, the critical causes of these changes, as well as ways to eliminate them, have not been identified yet.

The study of aging processes has shown [23,26] that oxygen adsorption affects both the electrical properties and the PL intensity of the NBE and DLE bands. The surface of ZnO has at least two types of sites with active O_2_ adsorbing centers, where O_2_ adsorption is of a different nature. One type of site is characterized by the fact that the conversion of the surface from O–ZnO to HO–ZnO is very stable. Other sites are unstable and are the cause of the aging effect; therefore, the aging process is critically dependent on the predominance of various active adsorbing centers on the surface. It is shown that a decrease in the number of active oxygen adsorption sites due to the formation of an HO–ZnO surface promotes the stabilization of the ZnO surface and suppresses the aging effect [23]. 

In the present article, we also show that two different types of sites with active O_2_ adsorbing centers are present on the ZnO surface. Their activity can be passivated by hydrogen, but the stability of the sites after passivation is significantly different. The first type of surface site is rapidly depassivated, and the aging effect takes place, due to these centers. The second type of surface sites is passivated in the form of HO–ZnO. It was found for the first time that the ratio between the first and second types of sites depends on the thermal history of the sample. This provides a technological method for overcoming the effect of degradation of the properties of ZnO during aging. The presence of two different types of sites with active O_2_ adsorbing centers in the near-surface region, showing significantly different stability after hydrogen passivation during subsequent aging, is confirmed by the combined data of XPS, PL and the electrical characteristics. Additionally, a simple method for obtaining a stable passivated HO–ZnO surface by means of preliminary heat treatment is demonstrated.

## 2. Materials and Methods

In the present study, ZnO layers were synthesized onto glass substrates (microscope glass slides 75 mm × 25 mm × 1.4 mm) or polished silicon substrates (50 mm × 10 mm × 0.5 mm) by chemical bath deposition (CBD) method. Initially, the glass or silicon substrates were thoroughly cleaned with distilled water, and a thin seed layer of ZnO (~1–10 nm) was deposited on substrates by the sol-gel method and annealed at 450 °C. An equimolar solution of zinc nitrate hexahydrate Zn(NO_3_)_2_·6H_2_O (Sigma Aldrich, St. Louis, MO, USA) and hexamethylenetetramine (CH_2_)_6_N_4_ (Sigma Aldrich) in ultrapure water (18.2 MΩ·cm) was used as the growth solution. The substrates with the seed layer were vertically immersed in 200 mL of this growth solution in a 250 mL beaker. A solution with a concentration of 15 mM or 200 mM was used to obtain ZnO nanorod arrays or thin ZnO films, respectively. The synthesis of ZnO samples was carried out in a water bath at a temperature of 90 °C for 1 h. After the CBD synthesis, the obtained samples were washed thoroughly with distilled water in an ultrasonic bath and cut into small sizes for further investigations. 

Thermal annealing was carried out at atmospheric pressure in air in the temperature range from 100 °C to 450 °C, using a quartz tube furnace. Plasma treatment in the hydrogen atmosphere (H-treatment) was carried out in a quartz cylindrical reactor with an internal diameter of 30 mm. During the hydrogen plasma treatment, the RF generator frequency, RF source power and discharge pressure were maintained at 27.12 MHz, 15 W and 70 Pa, respectively. Plasma treatment was carried out at room temperature without intentional substrate heating; however, the temperature of the substrate during treatment in hydrogen plasma increased slightly, up to ~60 °C, due to RF absorption.

The morphology of the samples was investigated by a Quanta 200i 3D (FEI, Hillsboro, OR, USA) scanning electron microscope (SEM). X-ray diffraction (XRD) analysis was carried out by a MiniFlex diffractometer (Rigaku, Tokyo, Japan) using Cu Kα radiation. The Hall effect of studies of ZnO films was measured at room temperature by the Van der Pauw four-probe method, using HMS-3000 Hall Effect Measurement System (ECOPIA, Anyang, South Korea) with a 0.56 T magnet. The photoluminescence (PL) spectra were recorded at room temperature under 300 nm excitation by a Cary Eclipse spectrofluorimeter (Agilent, Santa Clara, CA, USA) in the range of 300–850 nm. XPS spectra were measured by a NEXSA X-ray Photoelectron Spectrometer (Thermo Scientific, Waltham, MA, USA). Raman spectra were taken by a NTEGRA Spectra (NT-MDT, Zelenograd, Russia) spectrometer with a 473 nm solid-state exciting laser. Absorption spectra in the 190–1100 nm range were measured with a Lambda 35 (Perkin Elmer, Waltham, MA, USA) UV-vis spectrophotometer.

## 3. Results and Discussion

**Figure 1:**
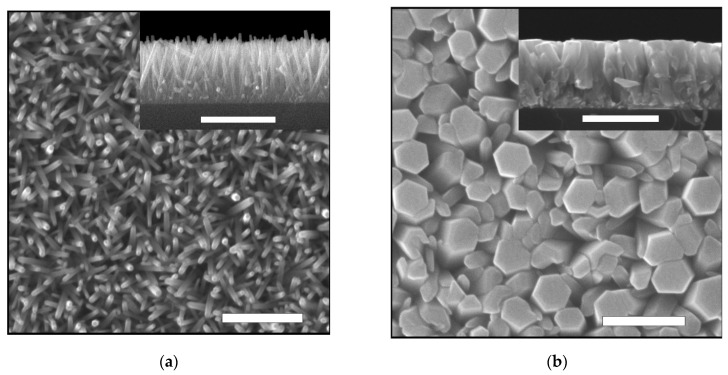

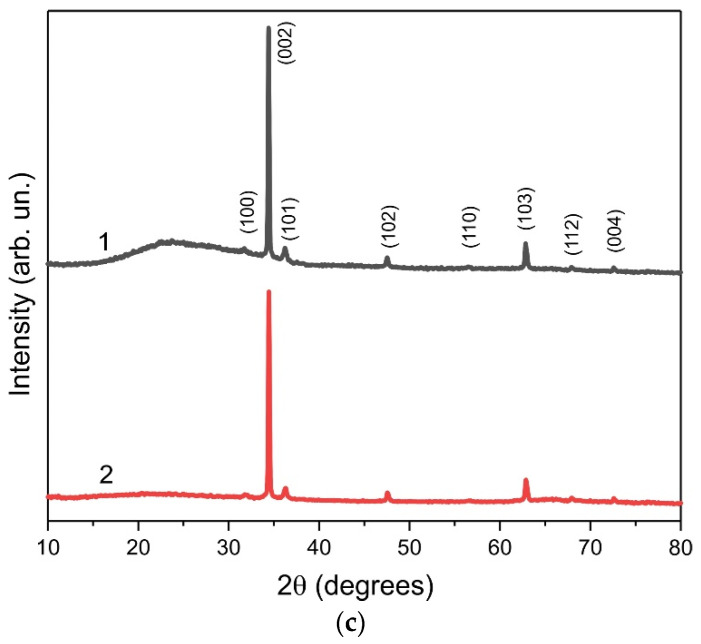
SEM images of ZnO layers synthesized by the CBD method at 90 °C for 60 min of (**a**) ZnO nanorods array grown in 15 mM solution and (**b**) ZnO films grown in 200 mM solution; the size of the images is 4 µm × 4 µm. The inset shows the cross-section of the samples. Scale bars = 1 µm; (**c**) XRD patterns of the obtained ZnO nanorods array (1) and ZnO film (2) on glass substrate.

The morphology of the synthesized ZnO samples depends on a number of growth process parameters, such as the temperature and concentration of the growth solution, and the synthesis duration. In particular, the layers of ZnO nanorod arrays were obtained at a low solution concentration (15 mM) at a synthesis temperature of 90 °C and synthesis time of 1 h (Figure 1a), and the ZnO films were synthesized at a concentration of 200 mM under the same conditions (Figure 1b). 

Figure 1c shows the XRD patterns of the as-grown (AG) ZnO samples synthesized on the glass substrates. Only XRD peaks of ZnO (JCPDS card # 01-089-0510) were detected, and therefore, both ZnO nanorods array and ZnO films consist of hexagonal wurtzite type structure with a preferred orientation along the (002) direction. The resistivity of the as-grown undoped ZnO films obtained by the CBD method was about ~100 Ω·cm, which is close to the literature data [12,18]. The low electrical conductivity of the as-grown polycrystalline ZnO films is mainly due to the surface effect.

As is known, various oxygen acceptors (O^−^, O^2−^, and others [6]) are present on the surface of grains. Since these oxygen-related acceptors capture free electrons from the bulk of the crystallites, the interfaces become negatively charged, and the bulk concentration of free carriers decreases. In addition, charged grain boundaries cause strong electron scattering; therefore, the carrier mobility is much lower in polycrystalline ZnO films than in ZnO single crystals.

**Figure 2:**
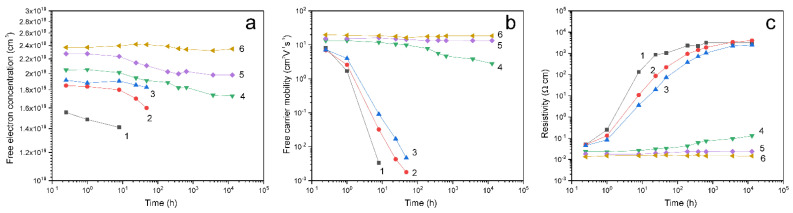
Concentration (**a**), mobility (**b**) and resistivity (**c**) of ZnO films: P-sample (1) and A + P samples annealed in air for 30 min at a temperature of 100 °C (2), 175 °C (3), 250 °C (4), 325 °C (5) and 400 °C (6) followed by treatment in hydrogen plasma for 3 min versus aging time.

To achieve low resistivity and high carrier mobility, surface acceptor passivation is necessary. It can be noted that the reverse process of depassivation is the cause of the aging effect and instability of properties. The passivation of surface acceptors was carried out in our case by short-term treatment of the as-grown ZnO samples in hydrogen plasma. It is well known that such treatment improves the electrical parameters of ZnO films [12]. Treatment in an RF plasma discharge in a hydrogen atmosphere for 3–4 min (such samples will be referred to as P-samples) led to a volume concentration of free carriers of ~1.6 × 10^19^ cm^−3^ (Figure 2). The resistivity of the films decreased to ~0.04 Ω·cm. The main reason for the decrease in the resistivity is the carrier mobility, which was ~8 cm^2^V^−1^s^−1^. The mobility increases due to a decrease in scattering at the grain boundaries as a result of hydrogen passivation of the acceptor traps on the ZnO polycrystals surface [27]. This H-treatment did not lead to any morphological or crystal changes in the samples that could be detected by SEM, or XRD.

**Figure 3:**
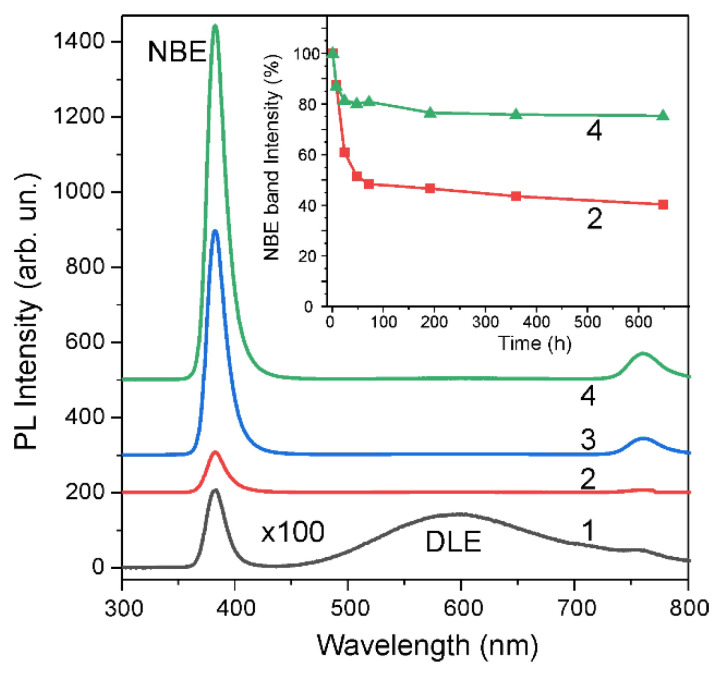
Room-temperature PL spectra of the as-grown ZnO (1), P-sample (2) and A + P samples preliminary annealed at 250 °C (3) or 400 °C (4) in air followed by H-treatment. Inset: dependence of the normalized NBE intensity for P-sample (2) and A + P sample (4) versus aging time.

The photoluminescence spectra of the AG samples also changed dramatically after H-treatment. The DLE band, which in as-grown samples had approximately the same intensity as the NBE band (Figure 3, spectrum 1), was completely passivated after H-treatment (Figure 3, spectra 2–4). The NBE band intensity in P-samples increased after H-treatment by a factor of ~50 as compared with the initial spectrum (Figure 3, spectrum 2). An increase in the PL intensity can be associated with the passivation of recombination centers on the surface and in the bulk, the introduction of shallow donors, etc. For example, specific chemisorbed oxygen and various radicals are present on the surface of ZnO obtained, using the CBD method; they significantly affect surface recombination and can be passivated by hydrogen [28,29,30].

It is found in the present work that the effect of activation of the electrical and PL properties due to H-treatment as well as the behavior of these properties during aging depends on the thermal history of the sample. Samples subjected to heat treatment in air will be referred to as A-samples, and samples subjected to heat treatment in air followed by H-treatment will be referred to as A + P-samples. The concentration and mobility of carriers in the A + P-samples increased with an increase in the temperature of preliminary isochronous (30 min) annealing (Figure 2), or with an increase in the duration of annealing at a fixed temperature (Figure S1 SUPPLEM). The concentration and mobility of carriers in A + P samples annealed at 400 °C increased to 2.4 × 10^19^ cm^−3^ and 20 cm^2^V^−1^s^−1^, respectively.

The PL spectra after H-treatment also demonstrate a strong dependence on preliminary annealing temperature. The A + P samples annealed at 250 or 400 °C followed by H-treatment showed an increase in NBE intensity by ~290 and ~460 times, respectively, in comparison with the initial PL spectrum (Figure 3). This strong dependence of the NBE intensity on the preliminary annealing temperature can be associated with a change in the structure of surface defects during annealing since it is known that the photoluminescent properties of nano- and microcrystalline ZnO are largely determined by the surface effect [13,14].

It is known that the adsorption of oxygen species causes the quenching of UV radiation of ZnO nanoparticles and the restoration of visible radiation [26]. However, a decrease in the NBE band intensity was not accompanied by an increase in the DLE band intensity in our A + P samples, and even the traces of the DLE band of visible radiation did not emerge upon aging. At the same time, upon aging of the P-samples, the DLE band appeared, although its intensity was very low. This indicates greater stability of hydrogen passivation in the A + P samples than in the P-samples.

The dependences of the electrical characteristics and PL intensity on the aging time allow us to conclude that there are two types of absorbing sites for O_2_ on the ZnO surface, where the stability of these absorbing sites under hydrogen passivation is significantly different. The first type is rapidly depassivated during aging; as a result, charged grain boundaries and surface acceptors, which play the role of carrier scattering centers and nonradiative recombination centers, are restored. The second type is characterized by high stability of the passivated state. The first type of absorbing sites for O_2_ dominates in as-grown samples, but when the preliminary annealing temperature increases, the fraction of the second type absorbing sites increases. 

XPS measurements confirm the presence of two types of absorbing sites for O_2_. A ~1 μm thick ZnO film grown by the CBD method on a silicon substrate was used in the XPS measurements. The film was divided into four samples, one of which was a control (AG–as-grown sample). The sample designated as A was annealed in air at 400 °C/20 min. The P-sample was plasma treated in a hydrogen atmosphere, under conditions given in the Experimental section above, for 3 min. The A + P sample was first annealed in air at 400 °C/20 min together with the A-sample and then H-treated for 3 min together with the P-sample. 

The XPS spectra of these four samples (AG, A, P, and A + P) were measured under the same conditions 6 days after H-treatment, i.e., the first XPS measurement was taken over >10^2^ h after H-treatment. During this time, the main aging processes of electrical parameters (Figure 2) and PL intensity (Figure 3) were completed; therefore, the XPS spectra should not contain adsorption centers, which are characterized by a rapid loss of the hydrogen passivation effect. The second XPS measurement was taken after another 70-day interval.

**Figure 4:**
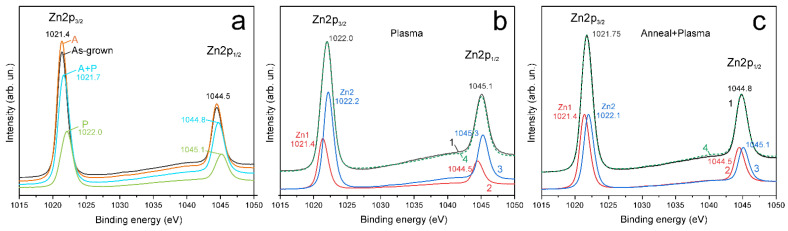
XPS Zn2p spectra of ZnO samples: (**a**) as-grown, annealed (A), H-treated (P), and annealed with subsequent H-treatment (A + P); (**b**) P-sample spectrum (black line); (**c**) A + P sample spectrum (black line); (**b**,**c**) also shown red line—Zn1 (2) and blue line—Zn2 (3) with relative intensities shown in Table 1, the green dashed line (4) on **b** and **c** is the sum of the Zn1 and Zn2 spectra.

XPS Zn2p core level lines: the XPS Zn2p spectrum of the AG sample (black line in Figure 4a) consists of two peaks Zn2p_3/2_ and Zn2p_1/2_; the spectrum corresponds to Zn^2+^ atoms (Zn–O) in the ZnO lattice. The distance between the peaks is ~23.1 eV, which is in good agreement with the known value of spin-orbit splitting of Zn2p in ZnO [31].

In what follows, the spectrum that is completely equivalent to the spectrum of the AG sample in terms of the shape, position, and full width at half maximum (FWHM) of the lines, but with a normalized intensity, will be denoted as Zn1. As can be seen from Figure 4a and Table 1, the position and FWHM of the Zn2p_3/2_ and Zn2p_1/2_ peaks in the A-sample are the same as in the AG-sample, although the XPS spectrum intensity increases slightly after annealing.

Plasma treatment shifts the Zn2p_3/2_ and Zn2p_1/2_ peaks toward higher energies. The FWHM of the peaks increases (Table 1), and the intensity of the peaks decreases. Possible explanations for the decrease in peak intensity are related to the surface potential [32] and the presence of a thin contaminating layer on the surface. However, the presence of a surface potential should cause a shift not only of the Zn lines, but also of the oxygen lines. As shown below, the oxygen lines are not shifted, and therefore, the surface potential cannot be the reason for the decrease in the intensity of the XPS spectra in the A + P and P-samples.

Since the decrease in the intensity of the XPS Zn2p_3/2_ and Zn2p_1/2_ lines (see Figure 4a) correlated with the increase in the C1s carbon line intensity, it can be assumed that one of the reasons for the decrease in the Zn2p spectra intensity is a contaminating carbon layer. The spectrum of the A-sample coincided with the AG-spectrum both in shape and in the peak position (Table 1), that is, the A-spectrum is proportional to the spectrum of Zn1.

It was found that the XPS Zn2p spectra of P and A + P samples can be very accurately represented by the sum of the spectrum Zn1 and same Zn1 but shifted in energy (spectrum Zn2), taken with certain coefficients. For example, the spectrum of the P-sample can be approximated as the sum of the Zn1 (red line in Figure 4b) and the Zn2, which is shifted in energy by +0.8 eV (blue line in Figure 4b); the intensity ratio of the Zn1 and Zn2 spectra should be 0.34: 0.66. The resulting total spectrum (green dashed line in Figure 4b) coincides very well with the spectrum of the P-sample. The same approximation is possible for the spectrum of the A + P sample; in this case, the shift of the Zn2 spectrum should be +0.7 eV, and the intensity ratio of the Zn1 and Zn2 peaks should be 0.50: 0.50. It can be seen that the total spectrum (green dashed line in Figure 4c) also coincides very well with the spectrum of the A + P sample. Thus, the XPS Zn2p spectra demonstrate that H-treatment transforms some Zn atoms in the near-surface region from the usual Zn1 state to the higher binding energy Zn2 states.

**Figure 5:**
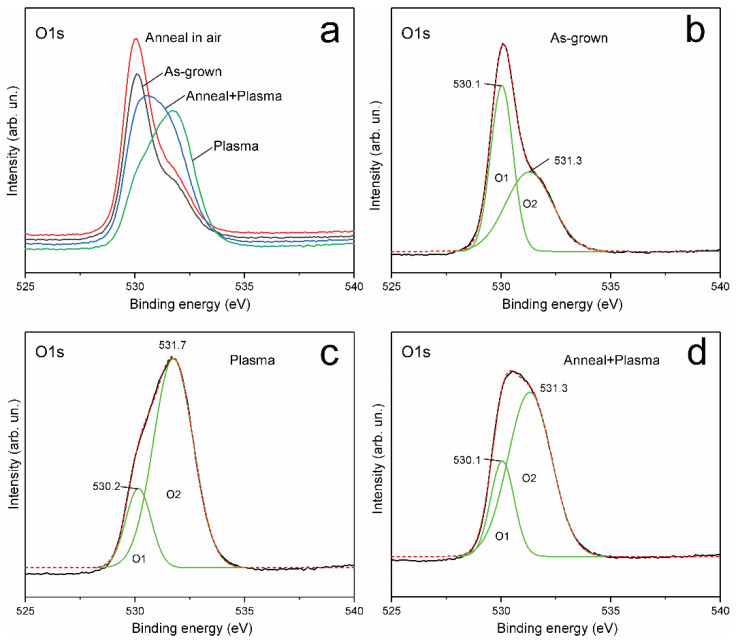
XPS spectra measured 6 days after plasma treatment: (**a**) XPS O1s spectra of AG, A, P and A + P samples; (**b**–**d**) XPS O1s spectra and deconvolution of the spectra (green lines) into two Gaussian peaks O1 and O2. The parameters of O1 and O2 peaks are given in Table 2; the dashed line corresponds to the sum of O1 + O2.

XPS O1s core level lines: the XPS O1s spectrum of the as-grown sample (Figure 5) is typical of ZnO grown by the CBD method and exhibits asymmetry, which indicates the presence of several forms of oxygen bonds in the ZnO near surface region. The XPS O1s spectra of all four samples can be deconvoluted into at least two peaks—O1 and O2 (Figure 5). Table 2 shows the position, FWHM, and relative contributions of the component peaks. The O1s peak with a maximum at ~530 eV (designated as the O1 peak) corresponds to O^2‒^ ions in the ZnO wurtzite structure. XPS O1s components with a higher binding energy of ~531.5 eV (designated as the O2 peak) correspond to O^2‒^ ions with a lower valence electron density and can be attributed to hydroxyls bonds, i.e., HO–ZnO, as well as to O^2‒^ ions in oxygen-deficient regions in the ZnO matrix [33].

As a result of annealing, the shape of the XPS O1s spectrum of the A-sample slightly changed in comparison with the spectrum of the as-grown sample (Table 2). In contrast, the XPS O1s spectrum of the P- and A + P samples changes significantly (Figure 5 and Table 2); the O2 peak intensity increases relative to the O1 peak as a result of H-treatment. The peaks Zn1 and O1 can be attributed to lattice zinc Zn^2+^ and oxygen O^2‒^ in wurtzite ZnO. The O2 peak in AG and A samples, whose contribution to the XPS O1s spectrum was ~56% (AG sample) and 50% (A sample), respectively (Table 2), cannot be associated with the Zn2 peak, which is introduced as a result of H-treatment since samples AG and A were not processed in hydrogen plasma. Note that the same effects, namely an increase in the energy of the XPS Zn2p_3/2_ peak, an increase of the higher energy component of the XPS O1s peak, the passivation of the DLE PL band, and the enhancement of the NBE PL band, were observed upon passivation of ZnO by SF6 plasma treatment [34].

As given in Table 1, in the XPS Zn2p_3/2_ spectrum, the Zn2 peak with a binding energy of 1022.2 ± 0.3 eV, which appears after H-treatment, has a binding energy ~ 0.8 eV higher than the Zn1 peak (1021.4 ± 0.2 eV), which corresponds to Zn^2+^ (Zn–O) atoms in the ZnO lattice. According to the literature, the Zn2 peak can be attributed to the more oxidized state of HO–ZnO [28,33]. It can also be expected that close binding energies will have Zn–O bonds on the surface, terminated by other radicals that are more electronegative than zinc, or with electronegativity comparable to that of hydrogen. Note that the XPS Zn2p spectrum does not contain components with a binding energy of less than 1022 eV, that is, H-treatment does not lead to the appearance of reduced zinc metal atoms on the surface. 

The XPS O1s components with a high binding energy of ~531.5 ± 0.3 eV (designated as the O2 peak) correspond to O^2‒^ ions with a lower valence electron density than O^2‒^ ions in ZnO and can be associated with several forms of oxygen. First, O2 components can correspond to non-lattice O^2‒^ ions or O^2‒^ ions in oxygen-deficient regions in the ZnO matrix (oxygen vacancies) [33]. In addition, the O2 peak can correspond to both pure hydroxyl bonds, that is, HO–ZnO [23], and hydrated oxides with differing degrees of hydration [35]. Surface-adsorbed weakly bound oxygen has close binding energy, about 532–533 eV, [23,32] and can also contribute to the O2 peak. The fact that the position and FWHM of the O2 peak in the XPS O1s spectrum slightly changes upon H-treatment indicates that the O2 peak consists of several contributions. 

The Zn2 components (i.e., HO–ZnO) in the XPS Zn2p spectrum are absent in the AG-sample and the A-sample; therefore, the oxygen-deficient regions make the main contribution to the O2 peak in the samples without H-treatment. The ratio of the O2/O1 peak areas can be used to estimate the relative amount of non-lattice oxygen [36].

As a result of hydrogen treatment, the Zn2 component appears in the XPS Zn2p spectrum; at the same time, the intensity of the O2 component of the XPS O1s spectrum significantly increases. Since plasma treatment in a hydrogen atmosphere causes reduction processes, the Zn2 peak and the gain of the O2 peak can be attributed to the formation of OH bonds, associated with the same species, such as HO–ZnO. 

As can be seen from Figure 4 and Figure 5, H-treatment significantly changes the XPS spectra of the samples and also introduces significantly more HO–ZnO sites into the AG-sample than into the preliminary annealed A-sample. This is easy to understand since the ZnO sample synthesized at a low temperature by the CBD method has a high concentration of surface defects; therefore, after H-treatment, the concentration of passivated states in the P-sample is high. Heat treatment of the AG-sample reduces the concentration of defects and transforms them into more stable configurations in the A-sample; accordingly, after the H-treatment, the concentration of passivated states in the A + P sample will be lower than in the P-sample. This conclusion is also confirmed by significant differences in the optical absorption spectra of the P-sample and the A + P sample (see the section on absorption, Figure S2 SUPPLEM).

**Figure 6:**
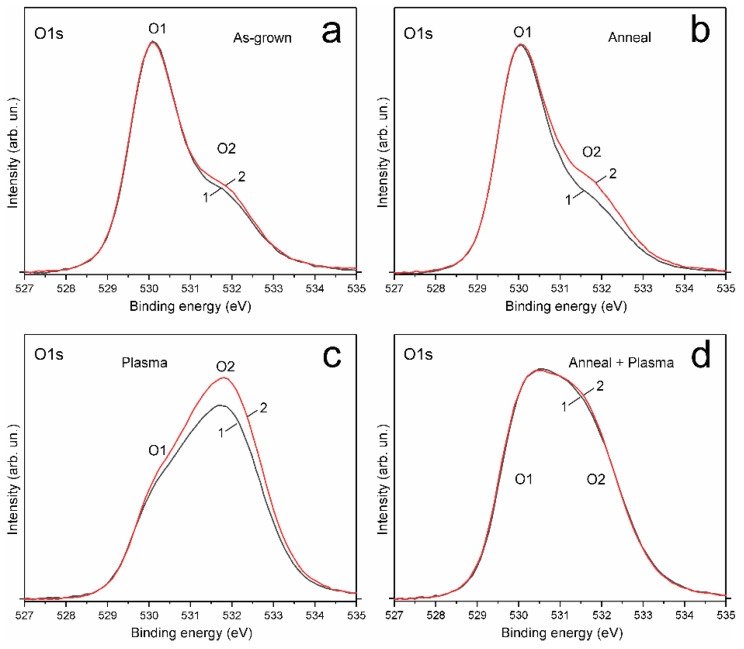
XPS O1s spectra of the ZnO samples measured 6 days (1) and 76 days (2) after plasma treatment: (**a**) AG sample, (**b**) A-sample, (**c**) P-sample, (**d**) A + P sample.

Repeated XPS measurements of the same samples were taken after 10 weeks, and these measurements did not reveal any noticeable changes in the XPS Zn2p spectra, compared to the first measurement, where the Zn1 to Zn2 peak intensity ratios and peak positions remained unchanged. At the same time, XPS O1s spectra showed noticeable changes (Figure 6). The intensity of the O2 peak increased in comparison with the O1 peak in the AG, A and P samples (Figure 6a–c), while the XPS O1s spectrum of the A + P sample did not change. Since the XPS Zn2p spectra did not change, the increase in the O2 peak intensity is not associated with HO–ZnO; this increase may be due to the adsorption of some other oxygen species. It is very important that the A + P sample spectrum demonstrates a very high stability during aging (Figure 6d), which indicates the stabilization of the surface after heat treatment followed by H-treatment. It can be concluded that the surface of AG, A and P samples is still capable of accepting new oxygen species; as a result, the degradation of properties takes place. At the same time, the surface of the A + P sample modified by thermal annealing is not capable of accepting new oxygen species, and such samples do not exhibit the aging effect. Thus, the effect of hydrogen treatment on the properties of the ZnO surface substantially depends on the preliminary thermal modification of the surface.

**Figure 7:**
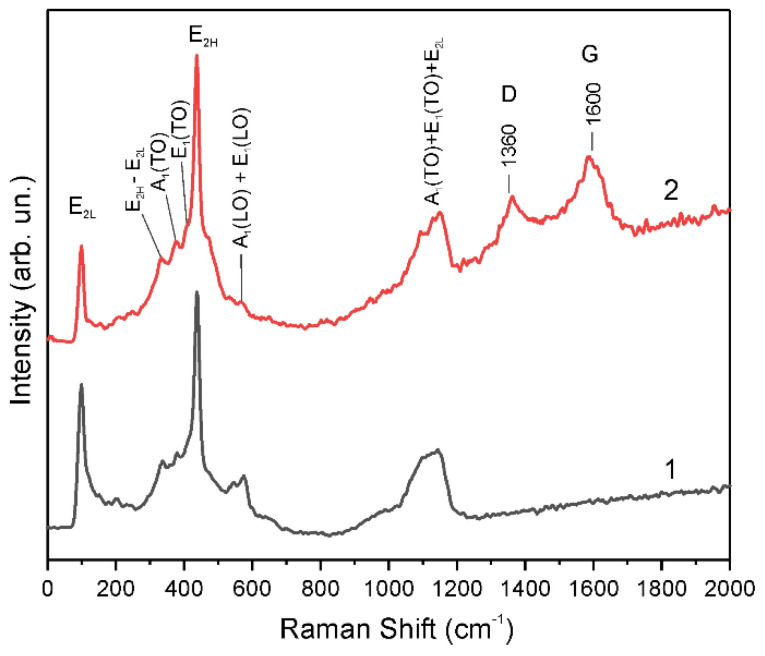
Raman spectra of two ZnO films simultaneously treated in hydrogen plasma for 3 min: (1) P-sample, (2) A + P sample.

Raman measurements were carried out to reveal the features of the surface properties of the samples annealed in air with subsequent hydrogen treatment. Two ZnO samples cut from the same glass substrate were used for these measurements, one of the samples was annealed in air at 400 °C for 30 min. Then, these samples were simultaneously processed for 3 min in hydrogen plasma at an RF generator power of 15 W. The Raman spectra of these two samples (P-sample and A + P sample) were measured under ambient conditions, using 473 nm excitation. The spectra of the samples in the range 100–1300 cm^−1^ showed characteristic ZnO modes [37] (Figure 7) and were practically identical. However, significant differences between the Raman spectra were found in the region from 1300 cm^−1^ to 1700 cm^−1^. The Raman bands of amorphous carbon, which are designated as D and G (Figure 7), appeared on the spectra of the A + P sample. At the same time, the P-sample that was treated in plasma without preliminary heat treatment did not show D and G bands.

The observation of carbon-related bands D and G shows that amorphous carbon, which is deposited during plasma H-treatment as a result of decomposition of residual organic contaminations, is formed only on the thermally treated surface of the ZnO films. Since the samples for such experiments were cut from the same substrate and were placed near each other during the H-treatment, the appearance of D and G carbon bands cannot be attributed to the different structures of the samples or different H-treatment conditions. Therefore, the samples subjected to preliminary heat treatment have increased catalytic surface activity during treatment with hydrogen plasma. To the best of our knowledge, this is the first report on the modulation of the catalytic activity of the ZnO surface by thermal annealing followed by hydrogen plasma treatment. 

The catalytic performance of the P and A + P samples was also studied by measuring the optical density spectra during the degradation of the Rhodamine B dye (RhB) on the ZnO surface under UV illumination. Figure S3a SUPPLEM shows the optical density spectra for the degradation of the RhB solution under UV radiation in the presence of the A + P sample, and Figure S3b shows the kinetics of RhB degradation as a function of the UV irradiation time. It can be seen that the activity of the A + P sample after annealing followed by hydrogen treatment is significantly higher than that of the P-sample subjected to plasma treatment only. This is consistent with Raman data on the increased catalytic surface activity of the A + P sample during hydrogen plasma treatment.

The high photocatalytic activity of the A + P sample can be explained by the higher optical absorption coefficient. The near band edge absorption spectra (Figure S2) show that the optical absorption values of AG, A and A + P samples were very close. A significant difference was observed only for the absorption spectra of the P-sample treated in hydrogen plasma (curve 2). The optical band gap was found to be E_g_ = 3.257 eV in the as-grown ZnO sample (Figure S2), 3.267 eV (P-sample), 3.250 eV (A-sample) and 3.262 eV (A + P sample). Attention is drawn to the fact that the absorption coefficient of P-sample at 3.3 eV was more than two times lower than in the AG, A and A + P samples. This suggests that the as-grown ZnO has a large number of electronic levels that are involved in the absorption of light, and the optical activity of these levels can be passivated by H-treatment. These levels are presumably surface states, because the electrical measurements show that the carrier mobility increases, i.e., the concentration of charged surface acceptor centers decreases as a result of the H-treatment.

## 4. Conclusions

The passivation of charged oxygen-related acceptors at the surface of grain boundaries makes it possible to activate the conductivity and photoluminescence of ZnO obtained by the CBD method and to obtain conductive transparent films with intense PL. However, the effect of passivation is unstable, and the electrical properties of the ZnO samples degrade during aging under ambient conditions, due to the capture of oxygen by active adsorption sites and recovery of charged grain boundaries. As a result, the resistivity increases by an order of magnitude or more, which makes it difficult to use in instrument structures, such as sensors, detectors, and TCOs. The intensity of the near band PL also decreases significantly during aging, due to the partial recovery of recombination centers on the surface of the grains, which prevents the creation of stable optoelectronic structures. Aging is caused by the presence on the ZnO surface of two different types of sites with active O_2_ adsorbing centers. The first type of adsorption centers is characterized by a rapid loss in the hydrogen passivation effect; the second type is the centers, the passivation of which is very stable. The transformation of the first type of centers into the second one ensures the stability of the surface properties. It is shown that the XPS spectra of the stable centers after hydrogen passivation correspond to HO–ZnO; these include the XPS Zn2p_3/2_ peaks at 1022.2 ± 0.2 eV and Zn2p_1/2_ at 1045.2 ± 0.2 eV, as well as a part of the XPS O1s peak at 531.5 ± 0.3 eV. The first type of adsorption centers dominates in as-grown samples. The number of second type centers increases with an increase in the preliminary annealing temperature in the range of 150–450 °C or with an increase in the annealing time at a fixed annealing temperature. 

Thermal annealing of polycrystalline ZnO films at ~400 °C in air followed by H-treatment allows to obtain films with low resistivity and intense PL, the properties of which are stable under ambient conditions. It can be concluded that thermal annealing modifies the surface, and stable configurations of surface defects are formed. In turn, the passivated states of these defects are very stable. The detailed transformation of the surface microstructure during thermal modification requires further research. 

It is also shown that the catalytic properties of thermally modified ZnO are higher than those of the unmodified sample, as evidenced by the reactions of accelerated decomposition of organic matter on the surface of the A + P sample during plasma treatment and the higher activity of the sample during the decomposition of RhB, compared to those of the P-sample which is subjected to only hydrogen treatment. Further studies are needed to elucidate the detailed mechanism of the zinc oxide surface modification as a result of oxidative thermal annealing.

## Figures and Tables

**Table 1:** Parameters of XPS Zn2p spectra of the samples.

Sample	Zn 2p_3/2_		Zn 2p_1/2_		Height
	Center, eV	FWHM, eV	Center, eV	FWHM, eV	
AG-sample	1021.4	1.69	1044.5	1.73	
A-sample	1021.4	1.69	1044.5	1.73	
P-sample (Zn1 + Zn2)	1022.0	2.05	1045.1	2.07	
Zn1 (Red line in Figure 4b)	1021.4	1.69	1044.5	1.73	0.34
Zn2 (Blue line in Figure 4b)	1022.2	1.69	1045.3	1.73	0.66
A + P sample (Zn1 + Zn2)	1021.8	1.93	1044.8	2.02	
Zn1 (Red line in Figure 4c)	1021.4	1.69	1044.5	1.73	0.50
Zn2 (Blue line in Figure 4c)	1022.1	1.69	1045.1	1.73	0.50

**Table 2:** Parameters of XPS O1s spectra of AG, A, P and A + P samples.

Sample	O1			O2		
	Center, eV	FWHM, eV	Area, %	Center, eV	FWHM, eV	Area, %
As grown	530.0	1.16	44	531.2	2.74	56
Anneal	530.0	1.20	50	531.2	2.50	50
Plasma	530.2	1.37	20	531.7	2.13	80
Anneal + Plasma	530.1	1.26	24	531.3	2.33	76

## Data Availability

Not applicable.

## Supplementary Materials

The following are available online at https://www.mdpi.com/article/10.3390/ma14216535/s1, Figure S1: Concentration, mobility and resistivity of ZnO films annealed in air at 200 °C for 7 min, 30 min, 150 min, 12 h and 60 h followed by H-treatment in hydrogen plasma for 3 min versus aging time; Figure S2: Absorption spectra and Tauc plots of the films: as-grown ZnO, P-sample, ZnO annealed at 450 °C for 20 min and annealed at 450 °C for 20 min followed by H-treatment; Figure S3: Optical density spectra of Rhodamine-B degradation under the UV radiation by A + P sample of ZnO film and the photocatalytic degradation of Rhodamine-B solution by P- and A + P samples as a function of UV irradiation time.
