# Supplementary material for: Stabilization of the Surface of ZnO Films and Elimination of the Aging Effect
Source: Materials (Basel). 2021 Oct 30;14(21):6535. doi: 10.3390/ma14216535 (PMC8585204; doi:10.3390/ma14216535)
Supplement: Supplementary file 1 [file materials-14-06535-s001.zip › materials-1401294-supplementary.pdf]

materials

MDPI

Supplementary materials

Stabilization of the surface of ZnO films and elimination of the aging effect

Khabibulla A. Abdullin [1,2,*], Maratbek T. Gabdullin [3], Sultan K. Zhumagulov [1], Guzal A. Ismailova [1], Lesya V. Gritsenko [4], Yevgeniya Y. Kedruk [4] and Mojtaba Mirzaeian [5]

1 al-Farabi Kazakh National University, al-Farabi ave. 71, 050040 Almaty, Kazakhstan; kh.abdullin@physics.kz (K.A.); zhumagulov.sultanbek@gmail.com (S.Z.); guzal_a81@mail.ru (G.I.)
2 Institute of Applied Science & Information Technology, Shashkin str., 40–48, 050040 Almaty, Kazakhstan
3 Kazakh-British Technical University, 050000 Almaty, Kazakhstan; gabdullin@physics.kz
4 Satbayev University, 050013 Almaty, Kazakhstan; gritsenko_lv@mail.ru (L.G.); y.kedruk@satbayev.university (Y.K.)
5 School of Computing, Engineering and Physical Sciences, University of the West of Scotland, Paisley, PA1 2BE, Scotland, UK; mojtaba.mirzaeian@uws.ac.uk
* Correspondence: kh.abdullin@physics.kz

Citation: Abdullin, K.A.; Gabdullin, M.T.; Zhumagulov, S.K.; Ismailova, G.A.; Gritsenko, L.V.; Kedruk, Y.Y.; Mirzaeian, M. Stabilization of the surface of ZnO films and elimination of the aging effect. *Materials* **2021**, *14*, 6535. https://doi.org/10.3390/ma14216535

Academic Editors: Cecilia Mortalò, Silvia Maria Deambrosis and Valentina Zin

Received: 14 September 2021
Accepted: 26 October 2021
Published: 30 October 2021

Publisher's Note: MDPI stays neutral with regard to jurisdictional claims in published maps and institutional affiliations.

Copyright: © 2021 by the authors. Licensee MDPI, Basel, Switzerland. This article is an open access article distributed under the terms and conditions of the Creative Commons Attribution (CC BY) license (https://creativecommons.org/licenses/by/4.0/).

ZnO layers were synthesized by the chemical bath deposition (CBD) method on glass or silicon substrates on which a ZnO seed layer (~ 1–10 nm) was preliminarily deposited by the sol-gel method followed by annealing at 450 °C. The CBD synthesis was carried out in an aqueous solution of zinc nitrate hexahydrate $Zn(NO_3)_2{\cdot}6H_2O$ (SigmaAldrich) and hexamethylenetetramine $(CH_2)_6N_4$ (Sigma Aldrich) in a water bath at 90 °C for 1 hour. A solution with a concentration of 15 mM or 200 mM was used to obtain arrays of ZnO nanorods or thin ZnO films, respectively. After the CBD synthesis, the obtained samples were thoroughly washed with distilled water in an ultrasonic bath and cut into small sizes for further studies. Thermal annealing was carried out in the temperature range from 100 °C to 450 °C in air. Plasma treatment in hydrogen atmosphere (H-treatment) was carried out at room temperature without intentional substrate heating. The RF generator frequency, RF source power and discharge pressure were maintained at 27.12 MHz, 15 W and 70 Pa, respectively, during the H-treatment.

To demonstrate that the rate of degradation of the electrical properties of ZnO films obtained in the course of low-temperature CBD followed by hydrogen plasma treatment strongly depends on the thermal history of the samples (on the temperature and duration of preliminary thermal annealing before hydrogen treatment), the electrical properties of ZnO films during aging were investigated. Figure S1 shows the change in concentration, mobility, and resistivity of ZnO films annealed in air at 200 °C for different durations between 7 minutes and 60 hours followed by 3 minutes of H-treatment. Note that these samples after annealing were H-treated simultaneously; therefore, the hydrogen passivation conditions for all samples were identical. Some difference in the parameters of the samples in Figure 3 (main text) and Figure S1 is caused by the fact that the samples were synthesized in different experiments. It can be seen that the longer the annealing time, the more stable the electrical properties during aging. These results confirm the conclusion that thermal annealing modifies the surface; stable configurations of defects on the surface are formed; and the passivated states of these stable defects are also stable and show no aging effect.

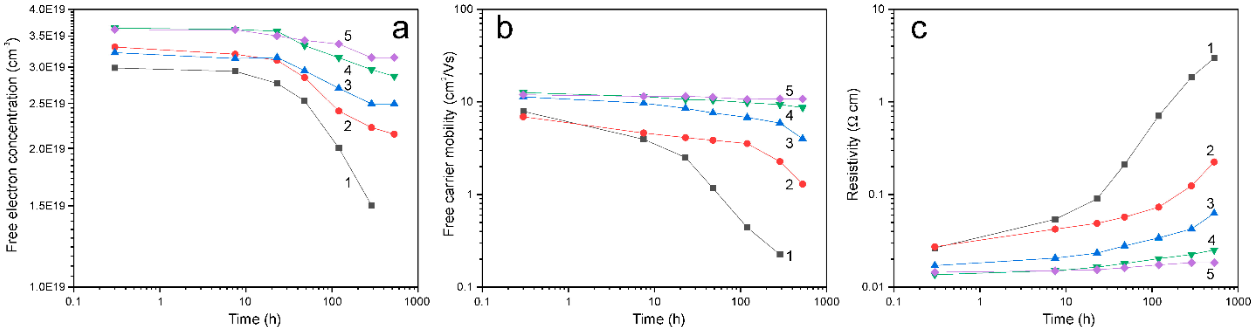

Figure S1. Concentration (**a**), mobility (**b**) and resistivity (**c**) of ZnO films annealed in air at 200 °C for 7 min (1), 30 min (2), 150 min (3), 12 hours (4) and 60 hours (5) followed by H-treatment in hydrogen plasma for 3 minutes versus aging time.

The near band edge absorption spectra also showed a dependence on the thermal history of the sample. The ZnO absorption spectra in the range from 300 nm to 1100 nm and the Tauc plot are shown in Figure S2. All these samples were cut from the same substrate and therefore, had the same thickness. The transmittance of these films in the visible range was 60–95%.

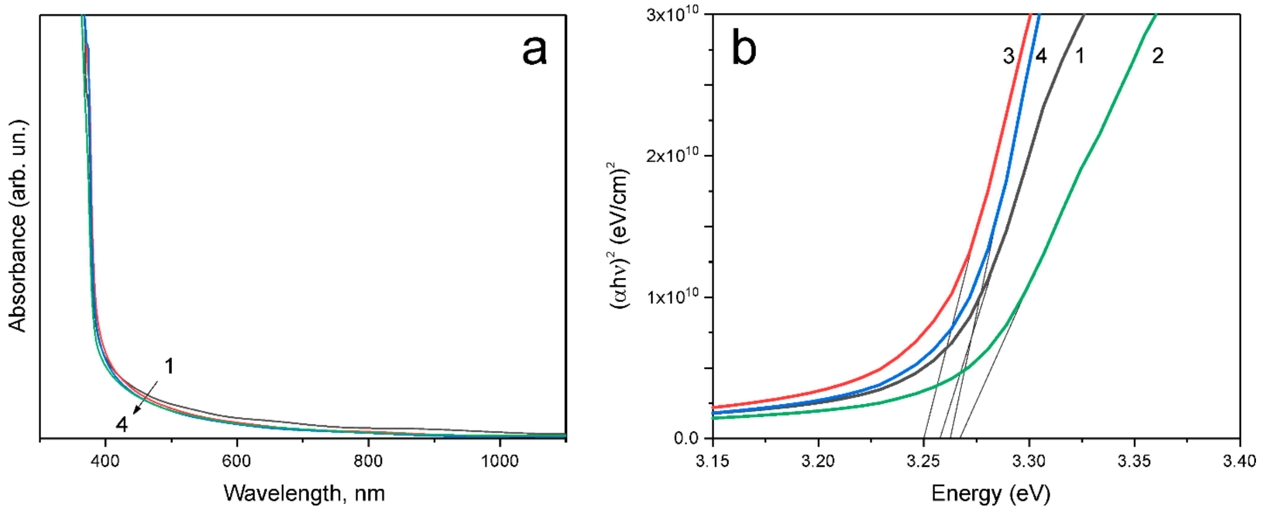

Figure S2. Absorption spectra (**a**) and Tauc plots (**b**) of the films: as-grown ZnO (1), P-sample (2), ZnO annealed at 450 °C for 20 min (3) and annealed at 450 °C for 20 min followed by H-treatment (4).

The optical absorption spectra near the band-gap of the as-grown sample and the sample annealed at 450 °C for 20 min (A-sample) as well as A + P sample were very close. A significant difference was observed only for the absorption spectra of the P-sample (Figure S2). The optical absorption spectra near the band-gap of the as-grown sample (Figure S2, curve 1) and the A-sample annealed at 450 °C for 20 min (curve 3) as well as the A+P sample annealed and treated in hydrogen plasma (curve 4) were very close. A significant difference was observed only for the absorption spectra of the as-grown sample treated in hydrogen plasma (P-sample, curve 2).

The optical band gap was found to be E_g = 3.257 eV in the as-grown ZnO sample (Figure S2b, curve 1), and E_g = 3.267 eV in the sample treated in H-plasma without preliminary annealing (Figure S2b, curve 2). Note that the absorption at a fixed energy, for example, at 3.3 eV, was significantly lower in the film subjected to H-treatment without preliminary annealing (Figure S2b, curve 2) than in the as-grown sample (curve 1) as well as in the samples annealed and treated in H-plasma (Figure S2b, curves 3 and 4). This suggests that the as-grown ZnO has a large number of electronic levels that are involved in the absorption of light, and the optical activity of these levels can be passivated by H-

treatment. These levels may be supposed to be surface states, because the electrical measurements show that the carrier mobility increases, i.e., the concentration of charged surface acceptor centers decreases as a result of the H-treatment.

The photocatalytic activity of the synthesized ZnO samples was measured in a glass flask with a volume of 250 ml on a magnetic stirrer. The Rhodamine B dye solution contained 0.16 g of RhB per liter of distilled water. The ultraviolet light was provided by a 14 W mercury arc lamp (LIH UL Q 14W 4P SE). The flask with RhB solution was cooled with running water. Samples of the RhB solution for measuring the optical density spectra were taken every 30 minutes for 2.5 hours.

Figure S3a shows the optical density spectra for the degradation of the RhB solution under UV radiation in the presence of A+P sample, and Figure S3b shows the kinetics of RhB degradation as a function of UV irradiation time. It can be seen that the activity of the A+P sample after annealing followed by hydrogen treatment is significantly higher than that of the P-sample subjected to plasma treatment only.

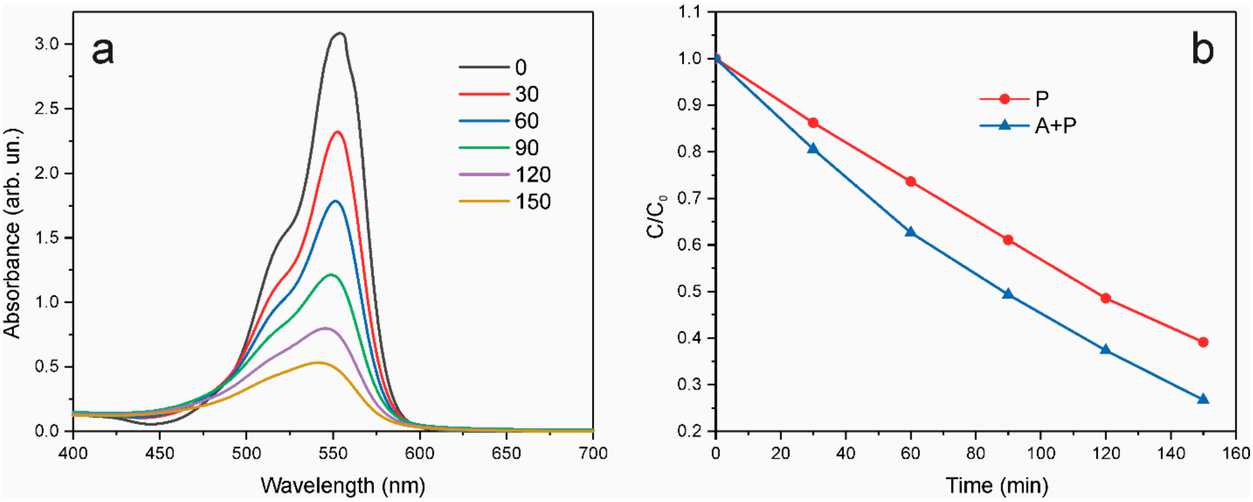

Figure S3. (**a**) Optical density spectra of Rhodamine-B degradation under the UV radiation by A+P sample of ZnO film; (**b**) the photocatalytic degradation of Rhodamine-B solution by P- and A+P samples as a function of UV irradiation time.
